# Association Between Cardiovascular Disease and Long-term Exposure to Air Pollution With the Risk of Dementia

**DOI:** 10.1001/jamaneurol.2019.4914

**Published:** 2020-03-30

**Authors:** Giulia Grande, Petter L. S. Ljungman, Kristina Eneroth, Tom Bellander, Debora Rizzuto

**Affiliations:** 1 Aging Research Center, Department of Neurobiology, Care Sciences and Society, Karolinska Institutet and Stockholm University, Stockholm, Sweden; 2 Institute of Environmental Medicine, Karolinska Institutet, Stockholm, Sweden; 3 Department of Cardiology, Danderyd Hospital, Stockholm, Sweden; 4 Environment and Health Administration, City of Stockholm, Sweden; 5 Stockholm Gerontology Research Center, Stockholm, Sweden

## Abstract

**Importance:**

Emerging yet contrasting evidence associates air pollution with incident dementia, and the potential role of cardiovascular disease (CVD) in this association is unclear.

**Objective:**

To investigate the association between long-term exposure to air pollution and dementia and to assess the role of CVD in that association.

**Design, Setting, and Participants:**

Data for this cohort study were extracted from the ongoing Swedish National Study on Aging and Care in Kungsholmen (SNAC-K), a longitudinal population-based study with baseline assessments from March 21, 2001, through August 30, 2004. Of the 5111 randomly selected residents in the Kungsholmen district of Stockholm 60 years or older and living at home or in institutions, 521 were not eligible (eg, due to death before the start of the study or no contact information). Among the remaining 4590 individuals, 3363 (73.3%) were assessed. For the current analysis, 2927 participants who did not have dementia at baseline were examined, with follow-up to 2013 (mean [SD] follow-up time, 6.01 [2.56] years). Follow-up was completed February 18, 2013, and data were analyzed from June 26, 2018, through June 20, 2019.

**Exposures:**

Two major air pollutants (particulate matter ≤2.5 μm [PM_2.5_] and nitrogen oxide [NO_x_]) were assessed yearly from 1990, using dispersion models for outdoor levels at residential addresses.

**Main Outcomes and Measures:**

The hazard of dementia was estimated using Cox proportional hazards regression models. The potential of CVD (ie, atrial fibrillation, ischemic heart disease, heart failure, and stroke) to modify and mediate the association between long-term exposure to air pollution and dementia was tested using stratified analyses and generalized structural equation modeling.

**Results:**

At baseline, the mean (SD) age of the 2927 participants was 74.1 (10.7) years, and 1845 (63.0%) were female. Three hundred sixty-four participants with incident dementia were identified. The hazard of dementia increased by as much as 50% per interquartile range difference in mean pollutant levels during the previous 5 years at the residential address (hazard ratio [HR] for difference of 0.88 μg/m^3^ PM_2.5_, 1.54 [95% CI, 1.33-1.78]; HR for difference of 8.35 μg/m^3^ NO_x_, 1.14 [95% CI, 1.01-1.29]). Heart failure (HR for PM_2.5_, 1.93 [95% CI, 1.54-2.43]; HR for NO_x_, 1.43 [95% CI, 1.17-1.75]) and ischemic heart disease (HR for PM_2.5_, 1.67 [95% CI, 1.32-2.12]; HR for NO_x_, 1.36 [95% CI, 1.07-1.71]) enhanced the dementia risk, whereas stroke appeared to be the most important intermediate condition, explaining 49.4% of air pollution–related dementia cases.

**Conclusions and Relevance:**

This study found that long-term exposure to air pollution was associated with a higher risk of dementia. Heart failure and ischemic heart disease appeared to enhance the association between air pollution and dementia, whereas stroke seemed to be an important intermediate condition between the association of air pollution exposure with dementia.

## Key Points

**Question:**

Does cardiovascular disease play a role in the association between long-term exposure to air pollution and dementia?

**Findings:**

In this cohort study of 2927 participants in the Swedish National Study on Aging and Care in Kungsholmen, air pollution exposure was associated with dementia risk despite comparatively low exposure levels. Heart failure and ischemic heart disease enhanced this association, and the development of stroke seemed to be an important intermediate condition.

**Meaning:**

In this study, virtually all of the association between air pollution and dementia seemed to occur through the presence or the development of cardiovascular disease, which suggests a need to optimize treatment of concurrent cardiovascular disease and risk factor control in older adults at higher risk for dementia and living in polluted urban areas.

## Teaser

This cohort study investigates the association of long-term exposure to air pollution with dementia and evaluates the role of cardiovascular disease in the association among participants of the population-based Swedish National Study on Aging and Care in Kungsholmen.

## Introduction

The number of people living with dementia is projected to triple in the upcoming 30 years.^1^ No curative treatment has been identified to date, and the search for modifiable risk and protective factors remains a clinical and public health priority.^1^

Research in the field of dementia has largely focused on the potential contribution of lifestyle factors, drug use, and health conditions.^1^ More recently, evidence concerning the association of exposure to air pollution and brain pathology is growing,^2^ in particular regarding neurodegeneration, excessive oxidative stress, and neuroinflammation.^3^ In addition, epidemiological and experimental evidence demonstrate an association of higher levels of pollutants in the air with faster cognitive decline.^4^ However, studies linking air pollution to dementia have sparse and inconsistent findings.

A number of studies on air pollution and cardiovascular health have identified air pollution as a risk factor for cardiovascular morbidity and mortality.^5,6^ Given the close association between cardiovascular burden and dementia,^7^ it is plausible to hypothesize that cardiovascular disease (CVD) might play a role in the association between air pollution and dementia.

The global exposure to ambient air pollution is substantial. Hence, even if air pollution is associated with modest-sized risks, reductions of population-level exposure may greatly affect the global burden of dementia. Shedding light on the mechanisms might further help in identifying vulnerable populations and prevention strategies.

We hypothesized that exposure to air pollution is associated with greater dementia incidence, and that CVD may mediate and strengthen that association. We aimed to test this hypothesis by using a well-characterized population-based cohort with spatially detailed data on long-term exposure to air pollution and longitudinal clinical assessments of dementia.

## Methods

We used data from the 2001-2013 Swedish National Study on Aging and Care in Kungsholmen (SNAC-K),^8^ a population-based longitudinal study. Eligible participants were residents of the Kungsholmen district in central Stockholm from March 21, 2001, through August 30, 2004, and 60 years or older. At baseline, 5111 individuals were selected at random from 11 age cohorts, of whom 521 were not eligible (200 had died, 262 had no contact information, 32 had moved, 23 were not native Swedish speakers, and 4 were deaf). Of the remaining 4590 individuals, 3363 (response rate, 73.3%) were examined. Since then, this cohort has been followed up regularly (attrition rate, 6%-11%) every 6 years for the young-old participants (aged 60-77 years) and every 3 years for older participants (aged ≥78 years); mean (SD) follow-up for the cohort was 6.01 (2.56) years. Follow-up was completed February 18, 2013. All participants or a proxy (in the case of cognitively impaired persons) provided written informed consent. The Regional Ethical Review Board in Stockholm, Sweden, approved the protocols of the SNAC-K study. This study followed the Strengthening the Reporting of Observational Studies in Epidemiology (STROBE) reporting guideline.

### Data Collection

For the present analysis, we excluded 11 individuals with missing data at baseline, 240 with prevalent dementia, and 185 (younger, healthier, and more often still working) who participated only at the baseline assessment, leaving a sample of 2927 participants. Trained staff performed face-to-face interviews and clinical and laboratory examinations. Home visits were performed for those who were unable to come to the research center. Data on age, sex, educational attainment, and age at retirement were obtained from the participants through a personal interview.^8^ Highest level of education attained was categorized as elementary school, high school, or university or above. Socioeconomic position was derived from the longest-held occupation and categorized as blue collar worker, white collar worker, or entrepreneur. Early retirement was defined as retirement before 65 years of age. Data on smoking were categorized as current, former, or never. Body mass index was obtained by dividing participants’ weight in kilograms by their height in meters squared. Level of physical activity was based on a questionnaire assessing frequency and intensity. As a measure of global cognition, we used the Mini-Mental State Examination. We extracted DNA from peripheral blood samples and performed genotyping for the apolipoprotein E (*APOE*) alleles. Participants were categorized as ε4 or non-ε4 carriers.

Comprehensive interviews and examinations from physicians, results of laboratory tests, use of medications, and registers from the Swedish National Patient Register were used to define diseases^9^ in accordance with the *International Statistical Classification of Diseases and Related Health Problems, Tenth Revision*. We considered the following conditions: CVD (ie, ischemic heart disease, heart failure, atrial fibrillation, and stroke), depression, and cardiovascular risk factors (ie, hypertension, type 2 diabetes, and dyslipidemia).

### Dementia Diagnosis

The diagnosis of dementia was made in accordance with the criteria of *Diagnostic and Statistical Manual of Mental Disorders, Fourth Edition, Text Revision*,^10^ following a 3-step procedure. First, a preliminary diagnosis was made by the examining physician, followed by a second preliminary diagnosis by a reviewing physician also involved in the data collection. In case of disagreement between the first and second diagnoses, the final diagnosis was made by a neurologist who was external to the data collection. Alzheimer disease (AD) was diagnosed according to the National Institute of Neurological and Communicative Diseases and Stroke/Alzheimer’s Disease and Related Disorders Association criteria^11^; and vascular dementia (VaD), according to the National Institute of Neurological Disorders and Stroke and Association Internationale Pour la Recherché et l`Enseignement en Neurosciences criteria.^12^ To account for the dementia cases among those who died between the 2 follow-up evaluations, we supplemented the diagnosis of dementia with the Swedish National Cause of Death Register and clinical medical records.

### Air Pollution Assessment

We estimated air pollutants at the residential addresses of participants with dispersion modeling based on local emission inventories, detailed elsewhere.^13^ Briefly, annual mean air pollution levels from local sources were calculated using emission inventories describing traffic and nontraffic sources for 1990, 1995, 2000, 2005, and 2011. We used NO_x _levels as a proxy for exhaust emissions from road traffic, and the local contributions to PM_2.5_ consisted of combustion particles from residential wood burning as well as exhaust and wear particles from road traffic. A gaussian dispersion model was applied to the emission databases together with meteorological and climate data. To allow high resolution in vicinity of roads, a quadtree receptor grid was used. Within the Kungsholmen district, 95% of the grid squares were 60 × 60 m^2^ or smaller. For streets flanked by buildings on one or both sides, an additional concentration component was simulated with the Danish Operational Street Pollution model.^14^ Annual mean levels of PM_2.5_ and NO_x _for 1990 through 2011 were obtained from linear interpolation during the 4 years between each model simulation, and levels for 2012 and 2013 were set as of 2011. To obtain total levels, annual long-range contributions, homogeneous across the model domain, were added to the simulated locally generated PM_2.5_ and NO_x_ levels, based on measurements at the rural site Norr Malma, located outside the calculation domain (60 km northeast of Stockholm). The total levels of PM_2.5_ are dominated by the contribution from long-range transport, resulting in a small relative spatial variance of the modeled PM_2.5_ level. Comparing the model-calculated levels with yearly measurements at 3 curbside (traffic) monitoring sites and 1 urban background site in Stockholm City for 1990 through 2011, correlations of 0.97 for NO_x_ and 0.86 for PM_2.5_ were obtained (eFigure 1 in the Supplement).

### Statistical Analyses

Data were analyzed from June 26, 2018, through June 20, 2019. Cox proportional hazards regression models were used to estimate hazard ratios (HRs) and 95% CIs for dementia associated with time-varying 5-year mean PM_2.5_ and NO_x_ exposures. Individuals were considered at risk until dementia diagnosis, death, or end of follow-up. We used age as the time scale. Associations between PM_2.5_ and NO_x_ exposures and dementia, AD, and VaD diagnoses were analyzed separately, first assuming a linear relationship. In a second stage, to assess departure from a simple linear trend, we modeled the continuous exposure using restricted cubic splines with 3 knots (2 *df*) at fixed percentiles (10th, 50th, and 90th) of its distribution. Compatibility of the data with a curvilinear relationship is formally assessed by testing the hypothesis that the coefficient of the second spline is zero; that is, when the cubic spline transformation of the predictor does not improve the fit of the data.

We explored to what extent CVD had a role in the studied association by testing 2 different hypotheses, modification and mediation. First, we hypothesized that air pollution would have a different effect on dementia depending on the presence of CVD (effect modification). To test this hypothesis, we stratified the analyses by CVD occurring any time during the follow-up period. Second, we hypothesized that CVD could act as a mediator of the association between air pollution and dementia. The mediating association was analyzed through generalized structural equation models. The association among the exposure, mediators, and outcome was assessed using logistic regression. To approach a model of a chain of distinct events, we took into consideration the exposure to air pollution 5 years before baseline and the presence of potentially mediating diseases in the dementia-free population by excluding all those cases with CVD that occurred to 5 years before baseline. Finally, we assessed the incidence of dementia during the following 13 years.

Potential confounders were defined a priori and chosen based on literature review; these included age, sex, educational attainment, smoking, physical inactivity, socioeconomic status, early retirement, body mass index, depression, baseline Mini-Mental State Examination score, and cardiovascular risk factors. Time trend in the level of air pollution, for cases with dementia and participants who did not develop dementia, was taken into account adjusting for year of assessment, which refers to the last year of the exposure period. Interaction between age and *APOE* ε4 status with the pollutants was tested, and stratified analyses were performed to evaluate their potential modifier effect. Two-sided *P* < .05 indicated statistical significance.

### Sensitivity Analyses

A competing risk model was performed to estimate the association between exposure to air pollutants and dementia, considering death without dementia as a competing event. To account for missing data (8.4%), we obtained 5 partially imputed data sets, pooled together using the Rubin rule. All variables included in the main analyses were used in the imputation model. All statistical analyses were performed with Stata, version 15 (StataCorp LLC).

## Results

At the study entry, the mean (SD) age of the 2927 participants was 74.1 (10.7) years; 1845 (63.0%) were female and 1082 (37.0%) were male; and 471 of 2915 with available data (16.2%) had an elementary level of education or below. Older participants (aged ≥78 years) had a lower mean Mini-Mental State Examination score (difference, 1.8; 95% CI, 1.7-2.1), and a greater number of chronic diseases (difference, 0.47; 95% CI, 0.38-0.55; *P* < .001) (Table).

**Table.** Characteristics of a Dementia-Free Cohort, Overall and Stratified by Age Groups and Dementia at Follow-up

Characteristic	Participant Group^a^				
	All (N = 2927)	Age <78 y (n = 2034)	Age ≥78 y (n = 893)	No Dementia (n = 2563)	With Dementia (n = 364)
Baseline					
Female	1845 (63.0)	1201 (59.0)	644 (72.1)^b^	1582 (61.7)	263 (72.3)^b^
Male	1082 (37.0)	833 (41.0)	249 (27.9)	981 (39.3)	101 (27.7)
Age, mean (SD), y	74.1 (10.7)	68.3 (6.7)	87.1 (4.9)^b^	72.8 (10.4)	83.1 (7.4)^b^
Educational attainment					
Elementary school	471 (16.2)	217 (10.7)	254 (28.7)^b^	391 (15.3)	80 (22.3)^b^
High school	1464 (50.2)	986 (48.5)	478 (54.1)	1250 (48.9)	214 (59.6)
University	980 (33.6)	828 (40.8)	152 (17.2)	915 (35.8)	65 (18.1)
Ever smoker	1359 (46.9)	840 (41.6)	519 (59.2)^b^	378 (14.9)	45 (12.7)
High physical activity level	619 (24.3)	553 (29.1)	66 (10.2)^b^	591 (25.8)	28 (11.0)^b^
BMI, mean (SD)	25.5 (4.1)	26.1 (4.0)	24.2 (4.0)^b^	25.7 (4.1)	24.3 (4.4)^b^
*APOE* ε4 carrier	774 (28.7)	597 (30.9)	177 (23.2)^b^	658 (27.8)	116 (37.5)^b^
Hypertension	2042 (69.8)	1369 (67.3)	673 (75.4)^b^	1787 (69.7)	255 (70.1)
Atrial fibrillation	274 (9.4)	127 (6.2)	147 (16.5)^b^	226 (8.8)	48 (13.2)^b^
Heart failure	287 (9.8)	95 (4.7)	192 (21.5)^b^	223 (8.7)	64 (17.6)^b^
Ischemic heart disease	439 (15.0)	221 (10.9)	218 (24.4)^b^	352 (13.7)	87 (23.9)^b^
Stroke	208 (7.1)	102 (5.0)	106 (11.9)^b^	156 (6.1)	52 (14.3)^b^
Type 2 diabetes	259 (8.8)	178 (8.8)	81 (9.1)	218 (8.5)	41 (11.3)
Dyslipidemia	1429 (48.8)	1057 (52.0)	372 (41.7)^b^	1264 (49.3)	165 (45.3)
Depression and mood disorders	255 (8.7)	171 (8.4)	84 (9.4)	213 (8.3)	42 (11.5)^b^
MMSE score, mean (SD)	28.5 (2.3)	29.0 (1.6)	27.1 (3.0)^b^	28.7 (2.1)	26.7 (3.0)^b^
During follow-up					
Survival status (death)	1306 (44.6)	561 (27.6)	745 (83.4)^b^	956 (37.3)	350 (96.2)^b^
Incident dementia	364 (12.4)	125 (6.1)	239 (26.8)^b^	NA	NA
Alzheimer disease	218 (7.9)	79 (4.0)	139 (17.9)^b^	NA	193 (53.0)
Vascular dementia	70 (2.7)	27 (1.4)	43 (6.2)^b^	NA	63 (17.3)
NO_x_ within 5 y, mean (SD), μg/m^3^	25.9 (8.8)	25.2 (8.7)	27.4 (8.8)^b^	25.8 (9.0)	26.9 (7.3)^b^
NO_x_ at 6-11 y, mean (SD) μg/m^3^	33.3 (12.1)	32.5 (12.1)	35.1 (12.1)^b^	33.2 (12.4)	34.5 (9.9)
PM_2.5_ within 5 y, mean (SD), μg/m^3^	8.4 (0.7)	8.3 (0.7)	8.4 (0.8)^b^	8.3 (0.7)	8.5 (0.6)^b^
PM_2.5_ at 6-11 y, mean (SD), μg/m^3^	8.6 (0.7)	8.5 (0.7)	8.7 (0.7)^b^	8.6 (0.7)	8.6 (0.6)

Abbreviations: *APOE*, apolipoprotein E; BMI, body mass index (calculated as weight in kilograms divided by square of height in meters); MMSE, Mini-Mental State Examination; NA, not applicable; NO_x_, nitrogen oxide; PM_2.5_, particulate matter no greater than 2.5 μm.

^a^ Unless otherwise indicated, data are expressed as number (percentage) of participants. Data were missing for 12 participants (0.4%) for educational attainment, 31 (1.1%) for smoking status, 378 (12.9%) for physical activity, 231 (7.9%) for *APOE* ε4, 9 (0.4%) for MMSE score, 171 (5.8%) for Alzheimer disease, and 301 (10.3%) for vascular dementia.

^b^ *P* < .05.

**Figure 1.**
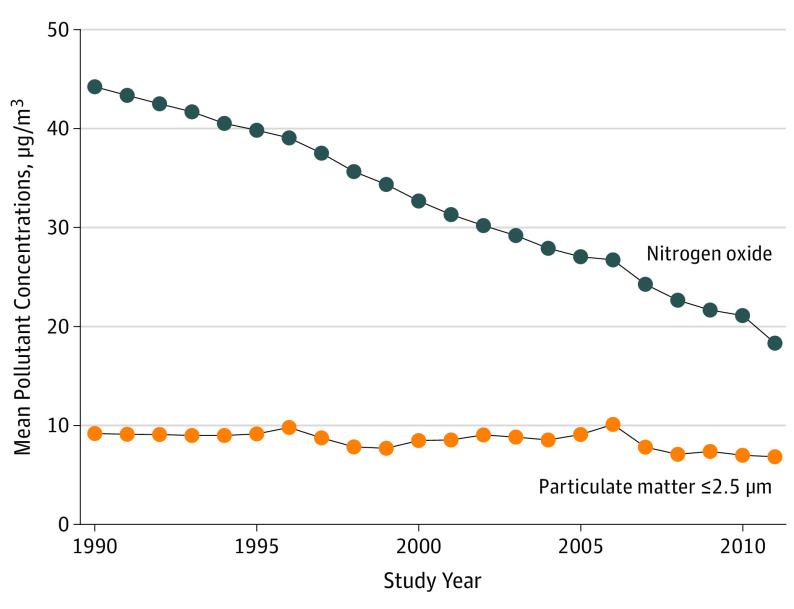
Trends in Modeled Concentrations of Residential Outdoor Pollution Levels of particulate matter of no greater than 2.5 μm and nitrogen oxide were measured for 21 years (1990-2011) as annual mean levels for the entire cohort (n = 2927).

Figure 1 shows the modeled annual mean concentrations of residential outdoor PM_2.5_ and NO_x_ for the cohort participants during the entire period (21 years). In the 5 years preceding the event, mean level for PM_2.5_ was 8.4 μg/m^3^ and for NO_x_ was 25.9 μg/m^3^; in the 6 to 11 years preceding the event, these levels were 8.6 μg/m^3^ and 33.3 μg/m^3^, respectively. A moderately high correlation was found between the 2 pollutants (Spearman correlation, 0.797; Pearson correlation, 0.854) in the 5 years preceding the event. Stronger correlations were found in the exposure time window 6 to 11 years preceding the event (Spearman correlation, 0.861; Pearson correlation, 0.930).

During follow-up, 364 incident cases were identified. Incident cases were more likely to be women (263 [72.3%]) and with a lower educational level (294 [81.9%]) and were a mean of 10.3 (95% CI, 9.1-11.4) years older at baseline than those who never developed dementia (*P* < .05).

A higher hazard of dementia was found per interquartile range (IQR) difference of PM_2.5_ (HR, 1.54; 95% CI, 1.33-1.78; IQR difference, 0.88 μg/m^3^) and NO_x_ (HR, 1.14; 95% CI, 1.01-1.29; IQR difference, 8.35 μg/m^3^) concentrations during the 5 years preceding the event, after considering potential confounders. Conversely, during the 6 to 11 years before the event, no association was found with PM_2.5_ (HR, 0.83; 95% CI, 0.71-1.01) or with NO_x_ (HR, 1.08; 95% CI, 0.96-1.22) concentrations. Concerning dementia subtypes, a higher hazard of VaD was found per IQR difference for PM_2.5_ (HR, 1.66; 95% CI, 1.38-1.99) and for NO_x_ (HR, 1.09; 95% CI, 0.98-1.30). No statistically significant associations were detected for AD.

**Figure 2.**
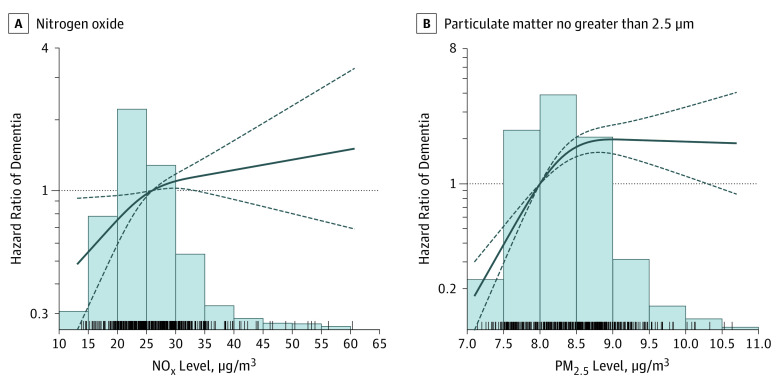
Hazard Ratios (HRs) of Dementia by Particulate Matter No Greater Than 2.5 μm (PM_2.5_) and Nitrogen Oxide Levels Estimates are HRs derived from Cox proportional hazards regression models. Air pollutants are modeled using restricted cubic splines. Age is considered as time scale. Models are adjusted for sex, age at baseline, year of assessment, educational attainment, smoking status, socioeconomic status, early retirement, physical activity, depression, baseline Mini-Mental State Examination score, type 2 diabetes, body mass index, hypertension, and dyslipidemia. The exposure period ranges from 0 to 5 years before the event. The reference group is considered the mean exposure level in the entire population. Bars represent distribution of the exposure levels in the entire population and spikes represent the cases. Solid line indicates point estimates; dashed lines, 95% CIs.

In the restricted cubic spline analysis, we observed a significant departure from linearity for both pollutants (PM_2.5_: Wald test, −4.54 [ *P* < .001] for second spline; NO_x_: Wald test, −1.43 [*P* = .04] for second spline). There was a strong increase in risk associated with exposure, from low to approximately mean levels, for both pollutants. Above the mean level, we observed no further increase in dementia hazard (Figure 2). The results coming from the restricted cubic splines analyses considering AD and VaD are reported in eFigure 2 in the Supplement.

**Figure 3.**
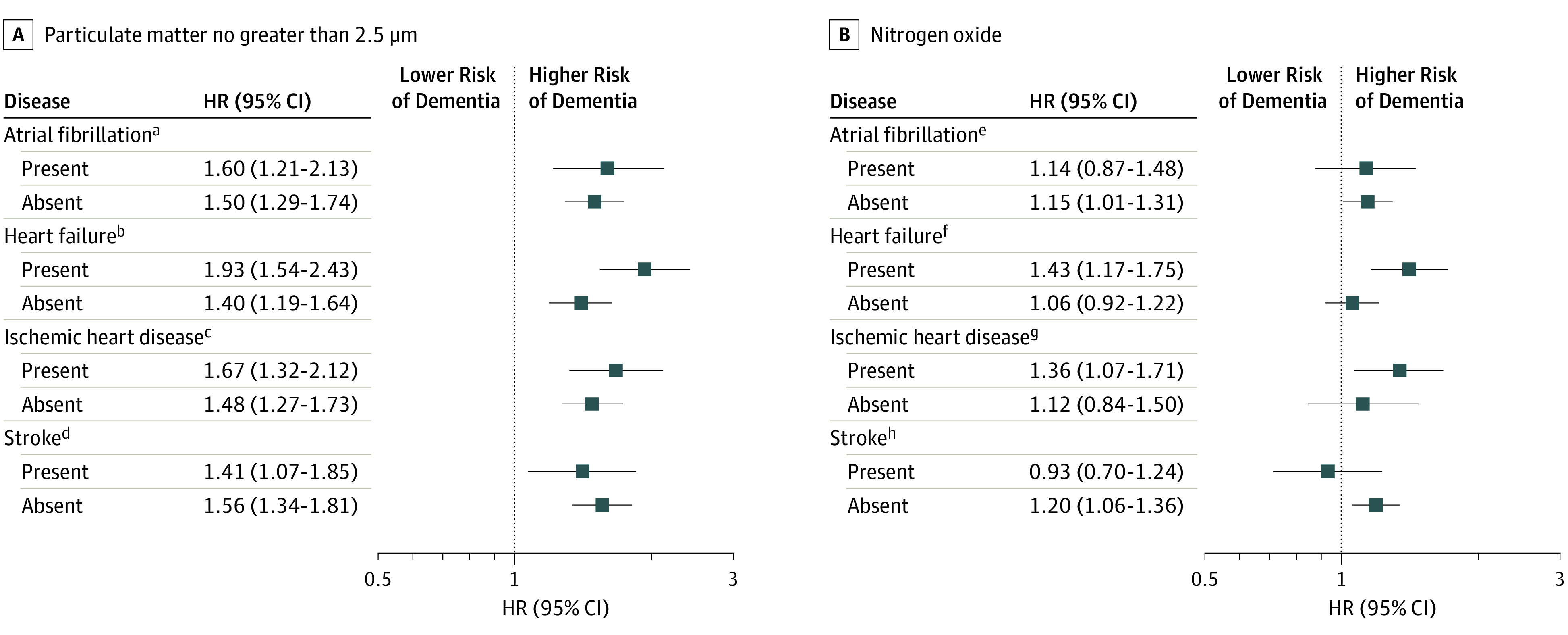
Hazard Ratios (HR) of Dementia by Particulate Matter No Greater Than 2.5 μm (PM_2.5_) and Nitrogen Oxide Stratified by Cardiovascular Disease Estimates are HRs derived from Cox proportional hazards regression models. Age is considered as time scale. Models are adjusted for sex, age at baseline, year of assessment, educational attainment, smoking status, socioeconomic status, early retirement, physical activity, body mass index, baseline Mini-Mental State Examination score, depression, hypertension, dyslipidemia, and type 2 diabetes. The time exposure period ranges from 0 to 5 years before the event. ^a^*P* = .67 for interaction. ^b^*P* = .001 for interaction. ^c^*P* = .37 for interaction. ^d^*P* = .50 for interaction. ^e^*P* = .94 for interaction. ^f^*P* = .02 for interaction. ^g^*P* = .13 for interaction. ^h^*P* = .10 for interaction.

In the stratified analyses by CVD, overall we observed a higher risk of dementia associated with exposure to PM_2.5_ and NO_x_ in persons with heart failure (interaction HR, 1.38 [95% CI, 1.06-1.81] and 1.35 [95% CI, 1.05-1.72], respectively) and (to a lesser degree) ischemic heart diseases (interaction HR, 1.13 [95% CI, 0.90-1.48] and 1.22 [95% CI, 0.95-1.60], respectively), in comparison with those without exposure (Figure 3). We did not observe any risk differences for stroke and atrial fibrillation.

**Figure 4.**
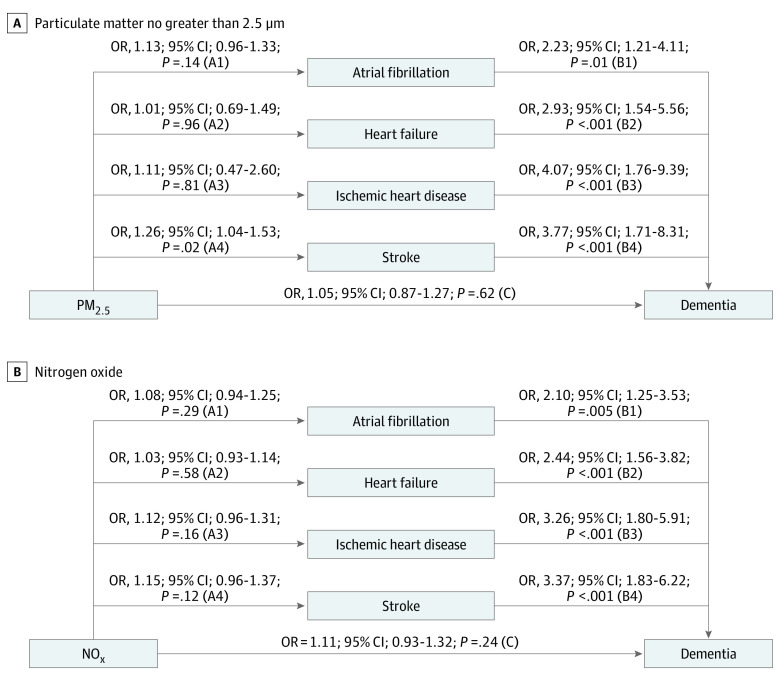
Association Between Levels of Particulate Matter No Greater Than 2.5 μm (PM_2.5_) and Nitrogen Oxide and Dementia Heart diseases and stroke were considered as mediators. Models are adjusted for sex, age at baseline, year of assessment, educational attainment, smoking status, socioeconomic status, early retirement, physical activity, baseline Mini-Mental State Examination score, body mass index, depression, hypertension, dyslipidemia, and type 2 diabetes. OR indicates odds ratio.

Figure 4 shows the path diagram between PM_2.5_ and NO_x_ levels and dementia, considering CVD at baseline as mediator. Higher levels of PM_2.5_ were associated with 75% increased odds (odds ratio, 1.75; 95% CI, 1.11-2.78) of dementia; higher levels of NO_x_ were associated with 66% increased odds (odds ratio, 1.66; 95% CI, 1.13-2.42) of dementia (total effect given by the sum of the β coefficients for the different paths: [A1 × B1] + [A2 × B2] + [A3 × B3] + [A4 × B4] + C). Half of the association between PM_2.5_ levels and dementia was explained by preceding stroke (49.4%; path A4 × B4). Higher levels of PM_2.5_ were found to be associated with 26% higher odds of stroke per IQR difference (path A4), and stroke was associated with a subsequent 3.8 times higher odds of dementia (path B4). Levels of PM_2.5_ were associated also with other potential CVD that all in turn showed strong associations with dementia (paths A1 × B1, A2 × B2, and A3 × B3). Only a fraction of the total association between PM_2.5_ levels and dementia was estimated to be through a direct effect (path C). No statistically significant mediation was found with CVD in the association between NO_x_ levels and dementia (Figure 4).

### Sensitivity Analyses

The competing risk regression model in which we considered dying without dementia as the competing event led to consistent results. Similar results were found in terms of direction and strength comparing the complete case analyses with the multiple imputation analyses. Stratified analysis showed similar results among individuals younger than 78 years and 78 years or older at baseline (PM_2.5_, Wald test, 5.96; NO_x_: Wald test, 1.81 [*P* > .15 for interaction]) and in *APOE* ε4 and non-ε4 carriers (PM_2.5_: Wald test, 0.30; NO_x_: Wald test, 0.36 [*P* > .29 for interaction]) (eFigure 3 in the Supplement).

## Discussion

In this large, population-based cohort study in a city with comparatively good ambient air quality, higher levels of exposure to air pollutants were associated with increased dementia incidence, and the last 5 years of exposure seemed the most important. Cardiovascular disease appeared to amplify the negative association of air pollution. In particular, heart failure and ischemic heart disease seemed to enhance the dementia risk, whereas stroke seemed to be an important intermediate condition between air pollution and dementia.

To date, only 1 recent Canadian study using administrative data has investigated the contribution of CVD in the association between air pollution and dementia, finding some evidence of an indirect effect through CVD for high levels of PM_2.5_ and NO_2_.^15^ Our findings add weight to this evidence by decomposing the indirect effect of single CVD and by testing the hypotheses of modification and mediation. Emerging evidence relates higher levels of air pollution to cognitive decline, pathological brain changes, and neurological hospital admissions.^4,16,17^ Interestingly, a handful of studies have also investigated the risk of dementia in association with air pollution. Our results are in line with those of Andersson and colleagues^18^ and Oudin and colleagues,^19,20^ who reported associations between NO_x_ and dementia in Northern Sweden among 1806 people followed up for 15 years. In a geographically diverse US cohort of 3647 women aged 65 to 79 years followed up for 10 years, Cacciottolo et al^17^ observed a 92% increased risk of all-cause dementia in those residing in areas where the US Environmental Protection Agency standards for PM_2.5_ were exceeded (>12 μg/m^3^) compared with low exposure levels. Finally, a large population-based study including all residents 55 years or older in Ontario^21^ showed a 7% to 11% increased dementia incidence in people living near major roads, even after adjusting for general background levels of PM_2.5_ and NO_x_. For a 5-year window, we observed a hazard of dementia per IQR difference increased as high as 50% in mean pollutant levels at the residential address. Notably, our results derive from a central area of Stockholm, where the control of environmental air pollution has been increasingly strict in the last decades. Interestingly, the higher limit reported herein is not only below the current European limit for fine particulate matter^22^ but also below the US standard. In other words, we were able to establish harmful effects at levels below current standards.

The biological mechanisms through which air pollution affects brain health are not completely understood, but several pathways are possible.^3^ Ultrafine particles and constituents of particulates may reach the brain via circulation and induce systemic inflammation, damaging the blood-brain barrier and activating the microglia.^3,23^ Also, animal data and postmortem studies indicate that particulates can be found in the olfactory bulb and the frontal cortical areas of brain of dogs and human beings with high levels of exposure to air pollution.^23^

Ambient air pollution could also affect the brain indirectly. Air pollution is an established risk factor for cardiovascular health, and it has been shown to be an important trigger of acute myocardial infarction,^24^ heart failure,^25^ and stroke.^26^ Because CVD accelerates cognitive decline and anticipates the onset of dementia,^7^ exposure to air pollution might negatively affect cognition, even without directly reaching the brain, by the detrimental effect of CVD. Notably, we were able to detect an increased risk of VaD, but not AD, with higher levels of air pollution, suggesting that a vascular component is at play in the development of dementia associated with air pollution. This impression is further supported by our mediation analysis, showing that most if not all association between air pollution and dementia could be explained by mediation through CVD, most notably stroke. However, results from previous studies^1,7^ showing mixed pathology in most of the dementia cases in older participants and the lack of biomarkers helpful in untangling the dementia etiology in the SNAC-K study suggest caution in the interpretation of results.

A number of possible mechanisms have been suggested to explain the association between air pollution and cardiovascular health. Air pollution has been reported to increase heart rate and decrease heart rate variability, increase blood viscosity, promote thrombus formation, and weaken atherosclerotic plaques.^27^ In line with these findings, our study further suggests that beyond the mediation through CVD, patients with heart failure and ischemic heart disease are at higher risk of developing air pollution–related dementia.

Several cohort studies have shown an association between long-term exposure to air pollution and cerebrovascular events.^28^ Stafoggia and colleagues^29^ reported a 19% higher risk of stroke for a 5-μg/m^3^ difference in PM_2.5_ levels. Besides the already mentioned damage to the vascular endothelium, the dysregulation of the sympathetic nervous system, and the accelerated atherosclerosis linked to air pollution, even moderate increases in PM_2.5_ levels have been associated with impaired cerebrovascular hemodynamics, including increased cerebral resistance and reduced cerebral blood flow.^30^

As already mentioned, PM_2.5_ and NO_x_ levels were associated with dementia risk. Notably, the estimates per IQR difference were larger for PM_2.5_, and findings from the mediation analysis for NO_x_ were not statistically significant. It should be noted that road traffic is a major local emission source for both pollutants, and the seemingly different effect between them should be interpreted with caution.

We observed a flattening of the effect size of dementia risk with increasing levels of pollutants; however, these findings should be interpreted with caution because the paucity of observations might be responsible for less precise estimates in these ranges. The exclusion of prevalent dementia cases might have led to a healthier and more resilient study sample, preventing us from detecting the expected higher risk of dementia in the older group. This might also explain why the presence of the *APOE* ε4 allele did not enhance the risk of dementia associated with air pollution.

### Strengths and Limitations

Major strengths of this study are the large population-based sample using a 13-year follow-up, with in-person extensive evaluations for clinical diagnoses of dementia and co-occurring chronic diseases. Most studies so far have used medical records that provide several benefits, including large, representative, and often nationwide samples. However, dementia is not always well captured in register-based studies, and mild cases are frequently overlooked,^31^ calling for detailed, high-quality clinical data.^32^ Notably, few studies considered death as a competing risk for dementia, but we identified dementia among participants who died during follow-up by reviewing clinical medical records and death registers, thus reducing the risk of death masking dementia. However, some milder cases might not have been captured, leading to a possible underestimation of the effect. Another major advantage is the assessment of air pollution from a highly detailed spatiotemporal model and covering a period starting 11 years before baseline.

Some limitations need to be acknowledged. The studied geographical area was urban only as it constitutes a part of the city of Stockholm. The spatial contrasts were thus limited (eg, reflected in the comparatively low IQR difference for PM_2.5_). In addition, because pollutants are correlated owing to common sources and spatial distributions, the associations we observe for one pollutant might be a proxy of another. Exposure assessment was based on residential address, thus ignoring exposure related to time spent in traffic while commuting to work, for example. However, previous studies in Stockholm have indicated that even in middle-aged persons, personal exposure to traffic-related air pollution was significantly associated with residential levels estimated in a similar way as in our study.^33^

## Conclusions

By 2050, 68% of the world population is expected to live in urban areas, where they are continuously exposed to air pollution. Together with the worldwide aging of the population, this poses global challenges when it comes to preventive strategies for dementia. Establishing and characterizing the association between air pollution and dementia has important consequences. We demonstrated an increased association between exposure to higher levels of air pollution and dementia, with stronger association for the last 5 years of exposure. From a policy point of view, this result is encouraging because it might imply that reducing air pollutant levels today could yield better outcomes already in the shorter term, reinforcing the need for appropriately set air quality standards. In our study, virtually all the association of air pollution with dementia seemed to be through the presence or the development of CVD, adding more reason to optimize treatment of concurrent CVD and risk factor control, particularly for people living in the most polluted areas of our cities.

## Notes

Supplement. **eFigure 1.** Comparisons Between Measured and Modeled Annual Mean Concentrations of Pollutants **eFigure 2.** Hazard Ratios (HR) of Alzheimer Disease and Vascular Dementia With 95% Confidence Intervals by PM_2.5_ and NO_x_ **eFigure 3.** Association Between Air Pollutants (NO_x_ and PM_2.5_) and the Hazard of Dementia
