# Supplementary material for: Association Between Cardiovascular Disease and Long-term Exposure to Air Pollution With the Risk of Dementia
Source: JAMA Neurol. 2020 Mar 30;77(7):801–9. doi: 10.1001/jamaneurol.2019.4914 (PMC7105952; doi:10.1001/jamaneurol.2019.4914)
Supplement: Supplement. — eFigure 1. Comparisons Between Measured and Modeled Annual Mean Concentrations of Pollutants eFigure 2. Hazard Ratios (HR) of Alzheimer Disease and Vascular Dementia With 95% Confidence Intervals by PM2.5 and NOx eFigure 3. Association Between Air Pollutants (NOx and PM2.5) and the Hazard of Dementia [file jamaneurol-77-801-s001.pdf]

Supplementary Online Content

Grande G, Ljungman PLS, Eneroth K, Bellander T, Rizzuto D. Role of Cardiovascular Disease in the Association of Long-term Exposure to Air Pollution and the Risk of Dementia. *JAMA Neurol.* Published online March 30, 2020. doi:10.1001/jamanetworkopen.2019.20548

eFigure 1. Comparisons Between Measured and Modeled Annual Mean Concentrations of Pollutants

eFigure 2. Hazard Ratios (HR) of Alzheimer Disease and Vascular Dementia With 95% Confidence Intervals by PM2.5 and NOX

eFigure 3. Association Between Air Pollutants (NOX and PM2.5) and the Hazard of Dementia

This supplementary material has been provided by the authors to give readers additional information about their work.

© 2020 American Medical Association. All rights reserved.

eFigure 1. Comparisons Between Measured and Modeled Annual Mean Concentrations of Pollutants

A. NO_x for 1990-2011

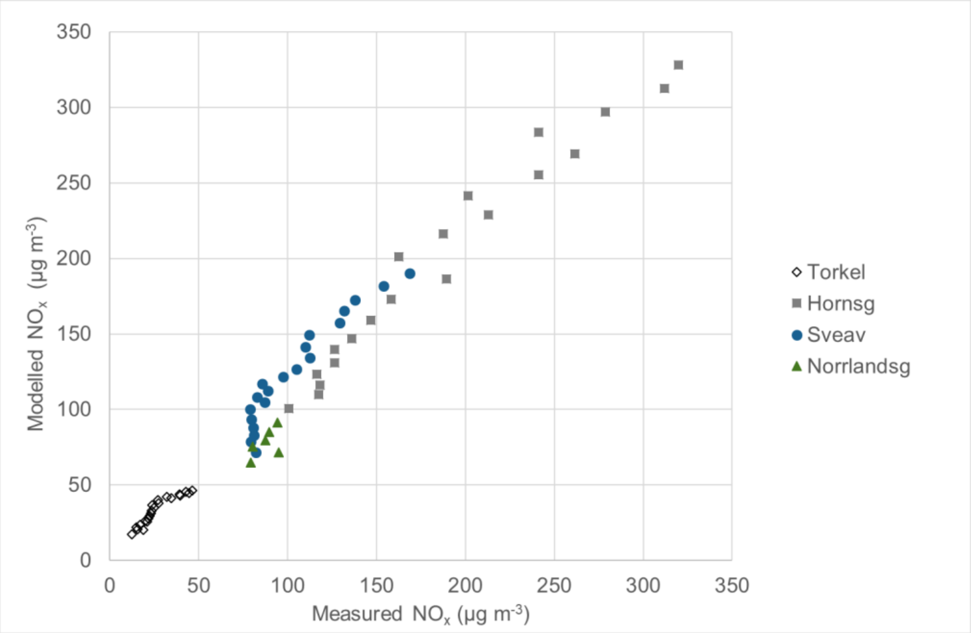

B. $PM_{2.5}$ for 1999-2011

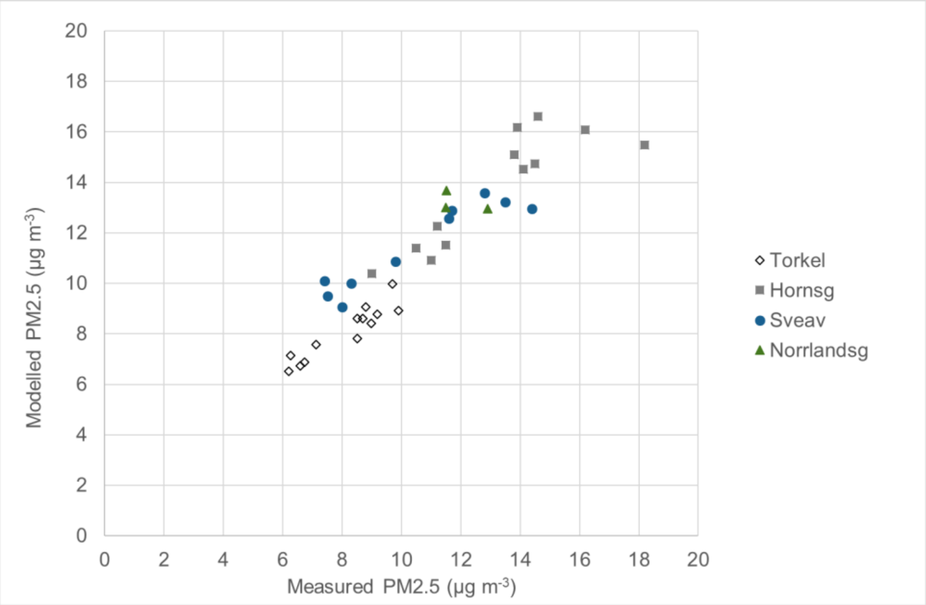

© 2020 American Medical Association. All rights reserved.

eFigure 2. Hazard Ratios (HR) of Alzheimer Disease and Vascular Dementia With 95% Confidence Intervals by $PM_{2.5}$ and NOX

Estimates are hazard ratios derived from Cox proportional hazard models according to $PM_{2.5}$ and NO_x levels. Air pollutants are modelled using restrict cubic splines. Age is considered as time scale. Models are adjusted for sex, age at baseline, year of assessment, education, smoking, socio-economic status, early retirement, physical activity, depression, baseline MMSE score, diabetes, BMI, hypertension and dyslipidemia. The time exposure period is between 0−5 years before the event. The reference group is considered the mean exposure level in the entire population. Bars represent distribution of the exposure levels in the entire population and spikes represent the cases.

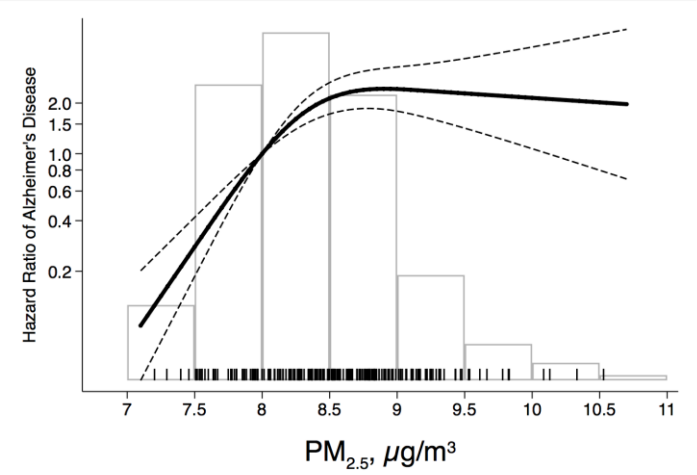

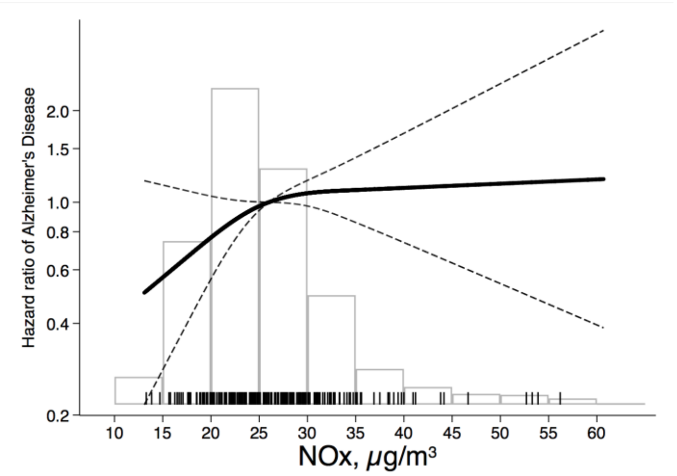

© 2020 American Medical Association. All rights reserved.

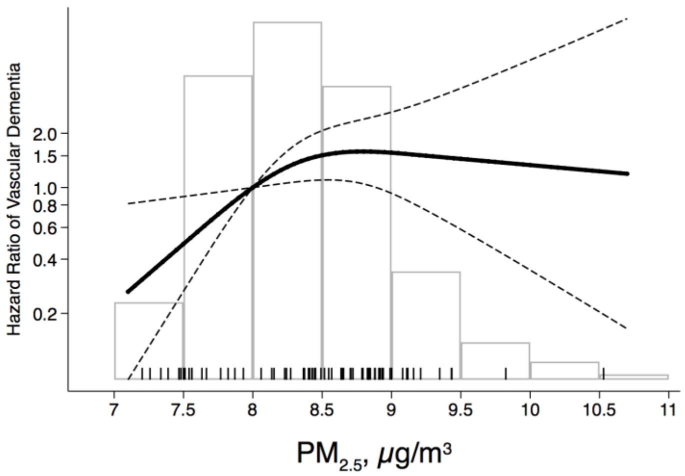

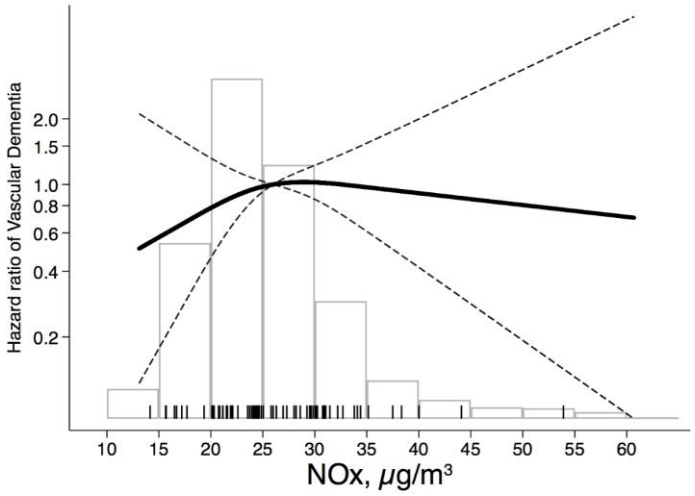

© 2020 American Medical Association. All rights reserved.

eFigure 3. Association Between Air Pollutants (NOX and $PM_{2.5}$) and the Hazard of Dementia

(A) Stratified by age groups: <78 years (blue line) and 78+ years (black line)

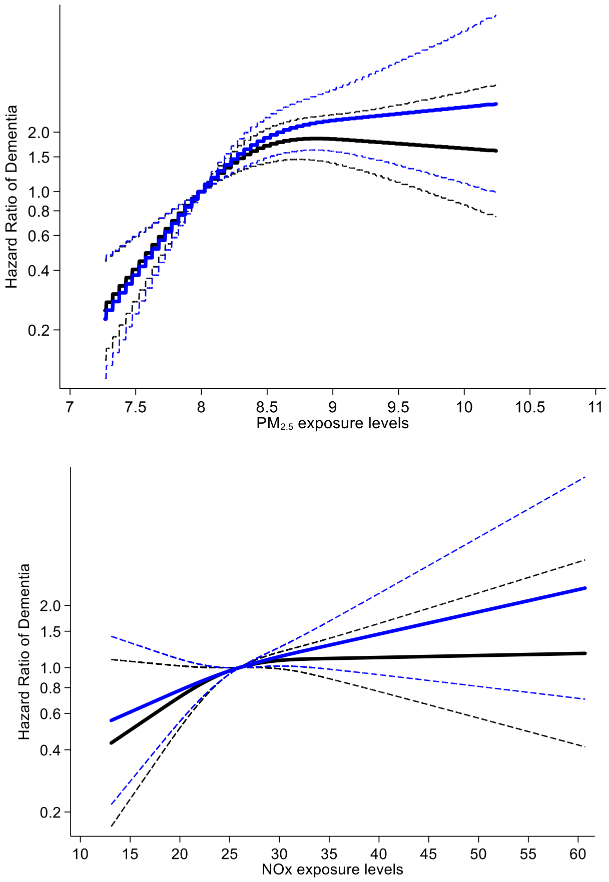

© 2020 American Medical Association. All rights reserved.

(B) Stratified by *APOE* status: at least one Ɛ4 allele (red line) and no Ɛ4 allele (black line)

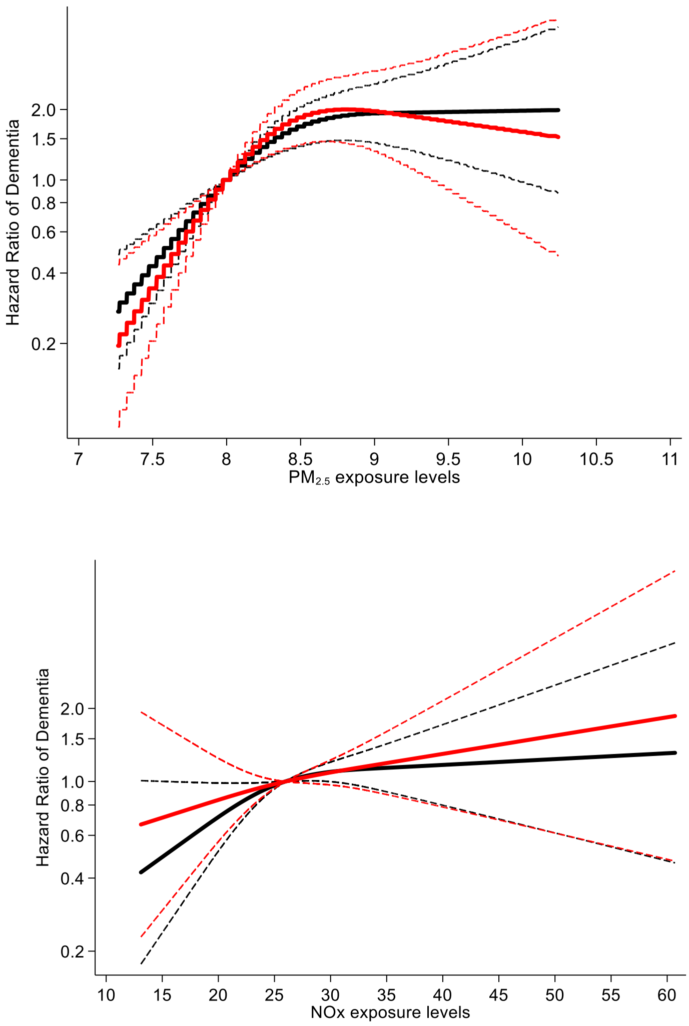

Estimates are hazard ratios derived from Cox proportional hazard models according to the air pollutants. Air pollutions are modelled using restricted cubic splines. Age is considered as time scale. Models are adjusted for sex, age at baseline, year of assessment, education, smoking, socio-economic status,early retirement, physical activity, baseline MMSE score, BMI, depression, hypertension, dyslipidemia and diabetes. The time exposure period is between 0−5 years before the event. The reference group is considered the mean exposure level in the entire population.

© 2020 American Medical Association. All rights reserved.
